# Phytochelatin Synthase has Contrasting Effects on Cadmium and Arsenic Accumulation in Rice Grains

**DOI:** 10.1093/pcp/pcx114

**Published:** 2017-08-14

**Authors:** Shimpei Uraguchi, Nobuhiro Tanaka, Christian Hofmann, Kaho Abiko, Naoko Ohkama-Ohtsu, Michael Weber, Takehiro Kamiya, Yuka Sone, Ryosuke Nakamura, Yasukazu Takanezawa, Masako Kiyono, Toru Fujiwara, Stephan Clemens

**Affiliations:** 1 Department of Public Health, School of Pharmacy, Kitasato University, 5-9-1 Shirokane, Minato-ku, Tokyo 108-8641, Japan; 2 Department of Applied Biological Chemistry, Graduate School of Agricultural and Life Sciences, The University of Tokyo, Yayoi, Bunkyo-ku, Tokyo 113-8657, Japan; 3 Department of Plant Physiology, University of Bayreuth, Universitätsstrasse 30, D-95440 Bayreuth, Germany; 4 Institute of Agricultural Science, Tokyo University of Agriculture and Technology, 3-5-8 Saiwaicho, Fuchu, Tokyo 183-8509, Japan

**Keywords:** Arsenic, Cadmium, Food safety, Metal accumulation, Metal tolerance, Rice

## Abstract

Phytochelatin (PC) synthesis has been well demonstrated as a major metal tolerance mechanism in *Arabidopsis thaliana*, whereas its contribution to long-distance element transport especially in monocots remains elusive. Using rice as a cereal model, we examined physiological roles of *Oryza sativa* phytochelatin synthase 1 (OsPCS1) in the distribution and detoxification of arsenic (As) and cadmium (Cd), two toxic elements associated with major food safety concerns. First, we isolated four different transcript variants of *OsPCS1* as well as one from *OsPCS2.* Quantitative real-time reverse transcription–PCR (RT-PCR) of each *OsPCS* transcript in rice seedlings suggested that expression of *OsPCS1full*, the longest *OsPCS1* variant, was most abundant, followed by *OsPCS2.* Heterologous expression of OsPCS variants in *PCS*-deficient mutants of *Schizosaccharomyces pombe* and *A. thaliana* suggested that OsPCS1full possessed PCS activity in response to As(III) and Cd while the activity of other PCS variants was very low. To address physiological functions in toxic element tolerance and accumulation, two independent *OsPCS1* mutant rice lines (a T-DNA and a *Tos17* insertion line) were identified. The *OsPCS1* mutants exhibited increased sensitivity to As(III) and Cd in hydroponic experiments, showing the importance of OsPCS1-dependent PC synthesis for rice As(III) and Cd tolerance. Elemental analyses of rice plants grown in soil with environmentally relevant As and Cd concentrations showed increased As accumulation and decreased Cd accumulation in grains of the T-DNA line. The *Tos17* mutant also exhibited the reduced Cd accumulation phenotype. These contrasting effects on As and Cd distribution to grains suggest the existence of at least partially distinct PC-dependent pathways for As and Cd.

## Introduction

Plants have transporter proteins for nutrient uptake from soil and distribution to various tissues. Some nutrient transporters also mediate acquisition of non-essential toxic elements such as cadmium (Cd) and arsenic (As) from soil. This is attributable to the chemical similarities between the essential and such non-essential elements and incomplete selectivity of nutrient transporters. As a consequence, plants accumulate Cd and As in their tissues. Phosphate transporter-mediated arsenate [As(V)] transport is a representative example (Shin et al. 2004, Wu et al. 2011, Kamiya et al. 2013). Arsenite [As(III)], another inorganic form of As, is an analog of silicic acid and dominant under the reducing conditions of paddy soil (Zhao et al. 2013). Silicon transporters and channels (Lsi1 and Lsi2) play a major role in As(III) uptake in rice roots (Ma et al. 2008). Plant Cd transport is mainly mediated by a variety of transporters for essential transition metals or cations (Krämer et al. 2007, Uraguchi and Fujiwara 2013). Especially in rice, a series of studies have identified transporters for Cd uptake (OsNramp5), vacuolar sequestration (OsHMA3), xylem loading in roots (OsHMA2) and distribution in shoots (OsLCT1) (Ueno et al. 2010, Miyadate et al. 2011, Uraguchi et al. 2011, Ishikawa et al. 2012, Sasaki et al. 2012, Satoh-Nagasawa et al. 2012). Knock-down or disruption of these transporter genes has achieved a reduction of As or Cd accumulation in rice plants. However, loss of transporter function can adversely affect elemental homeostasis and plant growth in some cases (Mitani-Ueno et al. 2011, Sasaki et al. 2012). Thus, it is important to explore further approaches suitable to ensure human-safe crop production.

Besides transporter proteins, metal ligands are another group of key players in metal transport and homeostasis. In plant cells, two metabolites, mugineic acid (MA) and its precursor nicotianamine (NA), form various metal complexes and facilitate iron (Fe) and zinc (Zn) transport (Haydon and Cobbett 2007, Kobayashi and Nishizawa 2012). Phytochelatins (PCs) represent another major type of metal-chelating ligand in plant cells. In contrast to MA and NA, PCs are predominantly associated with detoxification of non-essential toxic metals and metalloids. PCs are polypeptides with the general structure (γ-Glu-Cys)*_n_*-Gly (usually *n* = 2–7), and are non-ribosomally synthesized from glutathione (GSH) by phytochelatin synthases (PCSs). PCS-dependent PC synthesis in the cytosol is readily triggered by toxic metals and metalloids. In *Arabidopsis thaliana,* AtPCS1 is crucial for achieving tolerance against the toxic inorganic ions of Cd, As, Hg and Pb (Howden et al. 1995b, Ha et al. 1999, Fischer et al. 2014) as well as against Zn excess (Tennstedt et al. 2009). Another possible function of PCs is a contribution to long-distance element transport in plants. For example, PCs have been detected in the phloem of *Brassica napus* and implicated in Cd long-distance transport (Mendoza-Cozatl et al. 2008). Analyses of the AtPCS1-deficient *A. thaliana* mutant *cad1-3* and another allele, *cad1-6*, suggested PC-dependent Zn translocation from roots to shoots under adequate Zn condition (Kühnlenz et al. 2016). *cad1-3* also shows reduced root-to-shoot Cd translocation (Chen et al. 2006, Kühnlenz et al. 2016), but exhibits increased As translocation from the root (Liu et al. 2010). These findings in Arabidopsis suggest a potential of PCS to control Cd and/or As accumulation levels in seeds/grains. This question therefore needs to be addressed particularly in cereals.

However, few data derived from the analysis of mutant lines are available on the physiological roles of monocot *PCS* genes. There are precedent studies on rice *PCS* genes, but these only examined seed-specific knock-down of rice *PCS* genes (Li et al. 2007, Das et al. 2017). Only loss-of-function mutant analysis can unravel the contribution of PCSs to the mobility of toxic elements within rice plants*.* The significance of rice PCS has been strongly suggested by functional analysis of OsABCC1, a rice ortholog of the *A. thaliana* tonoplast ABC transporters for PC–metal(loid) complexes. OsABCC1 retains a substantial fraction of As in the vacuoles and thereby limits As movement within the plants and especially to the grains (Song et al. 2014).

A major motivation of the present study was to examine physiological roles of PCSs in Cd and As deposition to grains in rice as a cereal model as well as in Cd and As tolerance. We isolated cDNA of *OsPCS1* transcript variants and of *OsPCS2,* and conducted expression and functional analyses. We further identified two independent *OsPCS1* mutant rice lines and examined the physiological function of *OsPCS1* in Cd and As tolerance. Finally, the element accumulation was compared between the wild-type and *OsPCS1* mutant rice grown under hydroponic and soil culture conditions. Importantly, our data show contrasting effects of OsPCS1 on the accumulation of Cd and As in rice grains.

## Results

### Isolation and quantification of *OsPCS* transcripts

**Fig. 1:**
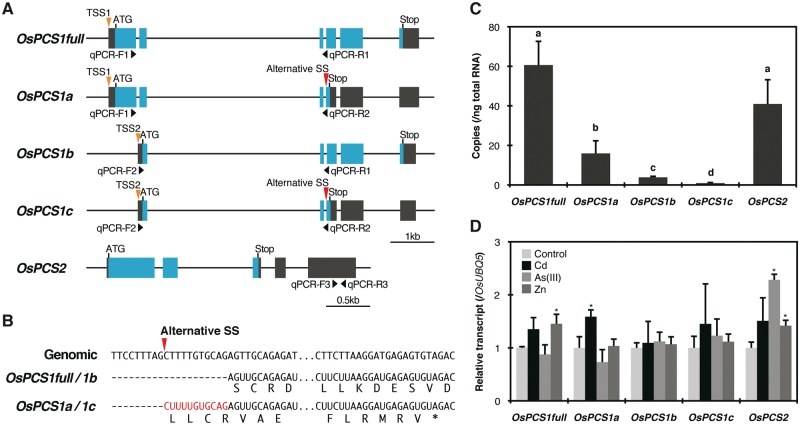
Isolation and characterization of *OsPCS* genes. (A) Predicted gene structures of *OsPCS1* (Os06g0102300/LOC_Os06g01260) and *OsPCS2* (Os05g0415200/LOC_Os05g34290). Black and blue boxes represent untranslated regions and coding regions, respectively. Approximate positions of real-time PCR primers, predicted transcription start sites (TSS1 and TSS2) and alternative splicing sites (SS) for *OsPCS1* variants are denoted with black, orange and red triangles, respectively. (B) Alternative splicing sites at exon 4/3 of *OsPCS1.* An additional 11 bp coding sequence for *OsPCS1a/1c* is indicated in red. (C) Absolute quantification of *OsPCS* transcripts in rice roots by real-time RT–PCR. Data represent means with the SD of at least three biological replicates. Means sharing the same letter are not significantly different (*P* < 0.05, Tukey’s HSD). (D) Responses of *OsPCS* transcripts in rice roots exposed to Cd, As(III) and Zn excess treatment for 3 h. Data represent means with the SD of at least three biological replicates. Asterisks indicate significant differences from control for each variant (*P < 0.05; *t*-test).

Two *PCS* genes have been found in the rice genome (Kühnlenz et al. 2014). Os06g0102300 (RAP-DB: http://rapdb.dna.affrc.go.jp/LOC_Os06g01260; MSU Rice Genome Annotation Project: http://rice.plantbiology.msu.edu) was predicted to encode the longest protein similar to AtPCS1. Thus the locus was named *OsPCS1,* and the second locus Os05g0415200/LOC_Os05g34290, which encodes a shorter protein, was referred to as *OsPCS2* (Kühnlenz et al. 2014). Because three different gene models in total were proposed for *OsPCS1* by the RAP and MSU databases, we verified the database predictions by isolating cDNA of *OsPCS1* transcript variants as well as of *OsPCS2* by PCR (Fig. 1A) and by examining their expression using specific primers for each variant (Fig. 1C). Four *OsPCS1* transcripts with different coding sequences were detected. The clones corresponding to LOC_Os06g01260.1, Os06t0102300-1/LOC_Os06g01260.2 and Os06t0102300-2 were named hereafter *OsPCS1full*, *OsPCS1a* and *OsPCS1b*, respectively. The *OsPCS1* transcript with the shortest coding sequence, not predicted in the databases, was named *OsPCS1c.* For *OsPCS1*, two different transcription start sites (TSS1 for *OsPCS1full* and *OsPCS1a* and TSS2 for *OsPCS1b* and *OsPCS1c*) were apparent as predicted in the databases (Fig. 1A). Furthermore, an alternative splicing site was indicated in exon 3 of *OsPCS1a/c*, which would add 11 bp to the start of exon 4 of *OsPCS1full/b* (Fig. 1B). This would result in an early stop codon for *OsPCS1a* and *OsPCS1c.* The *OsPCS2* sequence was also identical to that of the database. Compared with the length of PCS2 from other higher plants, the coding sequence of *OsPCS2* was shorter due to an early stop codon in exon 3 (Fig. 1A).

We performed quantitative real-time reverse transcription–PCR (RT–PCR) to verify the abundance of each *OsPCS* transcript (Fig. 1C). Six specific primers were designed and used in various combinations to distinguish all five *OsPCS* transcripts (Fig. 1A). Absolute quantification of *OsPCS* transcripts in rice roots demonstrated that *OsPCS1full* was the dominant molecule, followed by *OsPCS2.* The abundance of *OsPCS1a* was 25% of that of *OsPCS1full,* while the levels of the other *OsPCS1* transcripts were much lower. The public RNA-seq data available in the RAP-DB also indicated that *OsPCS1full* was the major *OsPCS1* variant in various rice tissues (Supplementary Fig. S1). Effects of Cd, As(III) or Zn excess treatment on the transcript abundance were not evident, except that As(III) treatment approximately doubled *OsPCS2* expression (Fig. 1D).

### Complementation analyses in fission yeast and *A. thaliana PCS* mutants reveal OsPCS1full as a functional PCS

**Fig. 2:**
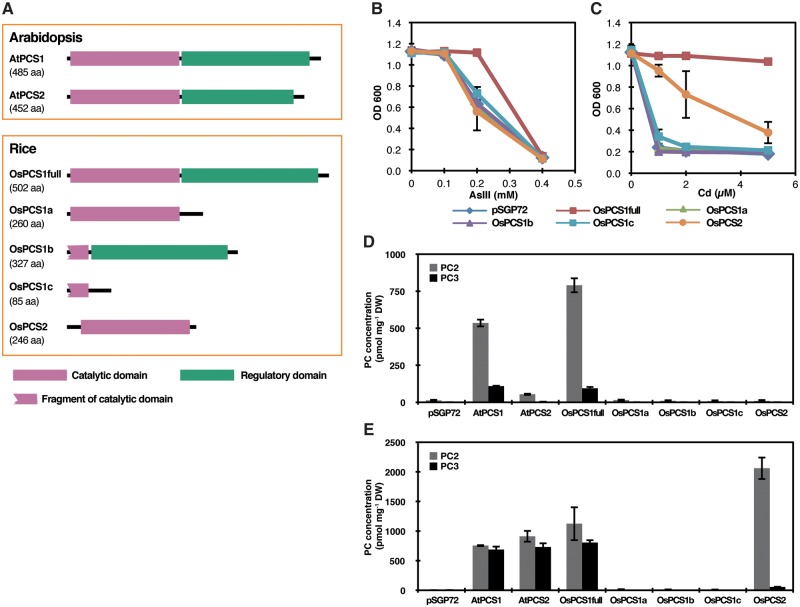
PCS activity of OsPCS variants heterologously expressed in *S. pombe Δpcs* under As(III) or Cd exposure. (A) Domain structures of AtPCSs and OsPCSs. The N-terminal catalytic domain (pfam05023) and C-terminal Phytochelatin_C domain of unknown function (pfam09328) are indicated. (B and C) Growth of *S. pombe Δpcs* harboring an empty vector pSGP72 or the cells expressing OsPCS variants when exposed to As(III) (B) or Cd (C). Data represent means with the SD of four independent replicates. (D and E) PC2 and PC3 concentrations in *S. pombe Δpcs* harboring an empty vector pSGP72 or the cells expressing AtPCS/OsPCS exposed to As(III) (D) or Cd (E). Data represent means with the SD of three independent replicates.

Based on the cDNA sequences, primary structures of the OsPCS proteins were compared with those of AtPCS1 and AtPCS2 (Fig. 2A; Supplementary Fig. S2). Both Arabidopsis PCSs have a structure canonical for higher plant PCS with two major domains (Rea 2012): the N-terminal catalytic domain (pfam05023) and the C-terminal Phytochelatin_C domain of unknown function (pfam09328). Among the predicted rice PCS proteins, OsPCS1full (accession No. LC314599) showed an AtPCS1/2-like structure with the two domains, whereas OsPCS2 (accession No. LC314600) would lack the C-terminal domain. OsPCS1a (accession No. AK071754) and OsPCS1b (accession No. AK071958) would consist of only the N- or C-terminal domain, respectively. OsPCS1c (accession No. LC314598) would contain a very short fragment of the catalytic N-terminal domain.

It was shown that C-terminally truncated AtPCS1 maintains substantial Cd-dependent PCS activity, suggesting that the N-terminal domain is sufficient for synthesis of PCs at least in response to Cd (Romanyuk et al. 2006, Kühnlenz et al. 2016). We therefore tested PCS activity of each predicted OsPCS using *Schizosaccharomyces pombe* as a heterologous expression system. The OsPCS1 variants and OsPCS2 as well as AtPCS1 and AtPCS2 were expressed in the *S. pombe* PCS knockout strain *Δpcs* (Clemens et al. 1999). Growth of the cells expressing OsPCSs was first tested under As(III) and Cd exposure. The cells expressing OsPCS1full showed better growth under 0.2 mM As(III) (Fig. 2B) and up to 5 µM Cd (Fig. 2C) than control cells with an empty vector pSGP72. OsPCS2 expression conferred weaker Cd tolerance but did not change As(III) tolerance. Growth of other cell lines was similar to that of the control cells under As(III) and Cd treatments. Subsequently, PC accumulation in the cell lines was quantified. Exposure to As(III) mainly induced drastic PC2 synthesis in the cells expressing OsPCS1full and AtPCS1 (Fig. 2D). In contrast, other OsPCS proteins including OsPCS2 did not show PCS activity. Cd treatment equally induced PC2 and PC3 synthesis mediated by OsPCS1full as well as by the two AtPCS proteins (Fig. 2E). In contrast to As(III), little but significant PC2 synthesis by OsPCS2 was observed in response to Cd exposure. However, far less PC3 was synthesized by OsPCS2.

**Fig. 3:**
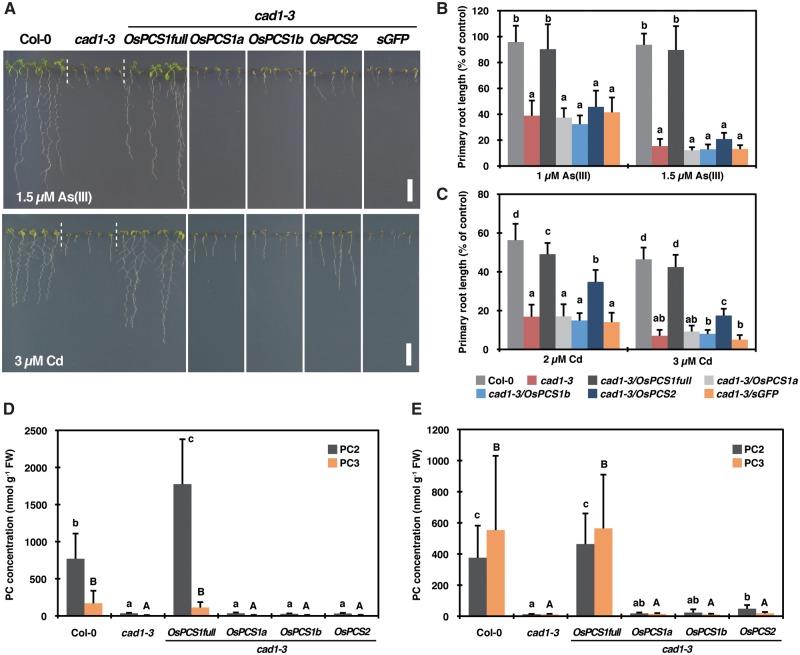
Complementation assay of *OsPCS* variants using the AtPCS1 null mutant *cad1-3.* (A) Phenotypes of Col-0, *cad1-3* and *cad1-3* transgenic plants expressing *OsPCS* variants or *sGFP* under control of the endogenous *AtPCS1* promoter grown on medium containing 1.5 µM As(III) or 3 µM Cd for 12 d. Scale bar = 1 cm. (B and C) Relative primary root length of Col-0, *cad1-3* and *cad1-3* transgenic plants grown on medium containing As(III) (B) or Cd (C) for 12 d. Data represent means with the SD of two independent experiments (*n* = 13–25). Means sharing the same letter are not significantly different within each treatment (*P* < 0.05, Tukey’s HSD). (D and E) PC2 and PC3 concentrations in roots of Col-0, *cad1-3* and *cad1-3* transgenic plants exposed to As(III) (D) or Cd (E). Seven-day-old seedlings were transferred from the control medium to the medium containing 5 µM As(III) or 5 µM Cd and grown for 7 d before harvest. Data represent means with the SD of two independent experiments (*n* = 3–4). Means sharing the same lower case (PC2) and upper case (PC3) letter are not significantly different (*P* < 0.05, Tukey’s HSD).

To examine further the PCS activity of predicted OsPCS proteins, in planta complementation was performed using the AtPCS1 null mutant *cad1-3* (Howden et al. 1995a). *cad1-3* plants were transformed with the vectors carrying the *AtPCS1* promoter and each *OsPCS* coding sequence except for *OsPCS1c.* The vector carrying *sGFP* fused with the *AtPCS1* promoter served as a control. Three independent T_3_ homozygous lines were established for each construct, and expression of the *OsPCS* variants was analyzed by RT–PCR (Supplementary Fig. S3). The transcript of the introduced *OsPCS* genes was evident in all *cad1-3/OsPCS* lines except for *cad1-3*/*OsPCS1b* line1. Growth of the plants on vertical agar plates was examined under control conditions and in the presence of As(III) or Cd. The concentrations were adjusted so that the stress severely inhibited the growth of *cad1-3* but had only a minor effect on Col-0 (Fig. 3A–C). In the absence of metal/metalloid stress, there was no difference in growth between the lines (Supplementary Fig. S4). The As(III)-sensitive phenotype of *cad1-3* seedlings was completely rescued by the introduction of *OsPCS1full*, whereas growth of the plants expressing other *OsPCS* genes was inhibited by As(III) as strongly as that of *cad1-3* (Fig. 3A, B). Similarly, *OsPCS1full* introduction complemented the Cd-sensitive phenotype of *cad1-3,* while other *OsPCS1* genes did not (Fig. 3A, C). In contrast to As(III), a partial rescue of the *cad1-3* phenotype upon Cd exposure was observed in the plants expressing *OsPCS2* (Fig. 3A, C). The phenotypes were consistently observed for all independent transgenic lines expressing a particular *OsPCS* gene (Supplementary Fig. S4). These results suggested OsPCS-mediated PC synthesis in (partially) complemented plants (*cad1-3/OsPCS1full* and *cad1-3/OsPCS2*) in response to As(III) or Cd.

To demonstrate such OsPCS-dependent PC accumulation in planta, PC2 and PC3 concentrations in the transgenic plants exposed to As(III) (Fig. 3D) or Cd (Fig. 3E) were measured. Wild-type-like PC accumulation was observed in plants expressing *OsPCS1full* under both As(III) and Cd exposure, whereas *OsPCS1a* and *OsPCS1b* expression did not enable PC synthesis. A slightly yet significantly elevated PC2 production was observed in Cd-treated *cad1-3/OsPCS2* compared with *cad1-3* (Fig. 3E). This trend was not observed under As(III) treatment (Fig. 3D). GSH concentrations in the plants were negatively correlated with PC concentrations (Supplementary Fig. S4): Col-0 and *cad1-3*/*OsPCS1full* contained less GSH compared with *cad1-3* and other transgenic lines under both As(III) (Supplementary Fig. S5A) and Cd exposure (Supplementary Fig. S5B). Taken together with the functional analysis in yeast, in planta complementation experiments showed that OsPCS1full is a fully functional PCS among rice PCSs in response to As(III) and Cd and OsPCS2 possesses a weak Cd-dependent PCS activity.

### *OsPCS1* mutant plants show increased sensitivity to Cd and As(III) stress

To understand the physiological roles of OsPCS1 in rice, *OsPCS1* mutant rice lines were identified (Supplementary Fig. S6A): a T-DNA insertion line (PFG_2D-20992, hereafter ‘T-DNA’ line) and three *Tos17* insertion lines (NG5039, NG5045 and NG5071). The *Tos17* insertion sites in the three *Tos17* insertion lines were confirmed at the same positon of *OsPCS1* intron 5 by sequencing. However, these lines appeared to be independent according to their respective secondary *Tos17* insertion sites in the genome according to the *Tos17* mutant panel database (NG5039, the long arm of Chromosome 7; NG5045, the long arm of Chromosome 6; NG5071, the long arm of Chromosome 12). RT–PCR analysis using primers amplifying a fragment spanning from exon 1 to 6 of *OsPCS1full* was conducted to estimate the expression level of *OsPCS1full* in each mutant. The transcript was not detected in the T-DNA line, while relative expression levels among the *Tos17* lines varied from 58% to 93% of cv. Nipponbare (hereafter, NB), the wild-type background of the *Tos17* lines (Supplementary Fig. S6B). NG5045 was selected for further experiments based on the lowest *OsPCS1full* expression among the tested *Tos17* lines. The second *Tos17* insertion of NG5045 is in an intron of a non-protein-coding transcript (Os06g0707800). Quantitative RT–PCR using the primers amplifying an upstream region of the *Tos17* or T-DNA insertion further confirmed the reduction of *OsPCS1full* expression by nearly 50% in NG5045 and down to residual level (<15%) in the T-DNA line, relative to NB and Hwayoung (hereafter HY), the wild type of the T-DNA line, respectively (Supplementary Fig. S6C). Thus, the T-DNA line represents a strong allele of *OsPCS1*, whereas NG5045 is a weak knock-down line.

The PC concentrations in the mutants exposed to Cd were measured to address whether the mutations affected PCS activity in these plants (Supplementary Fig. S6D). In accordance with the expression levels of *OsPCS1* in the mutants, total PC concentration was 60% of that of NB in NG5045 and 30% in the T-DNA line compared with HY. Reduction was more pronounced for PC3 than for PC2.

**Fig. 4:**
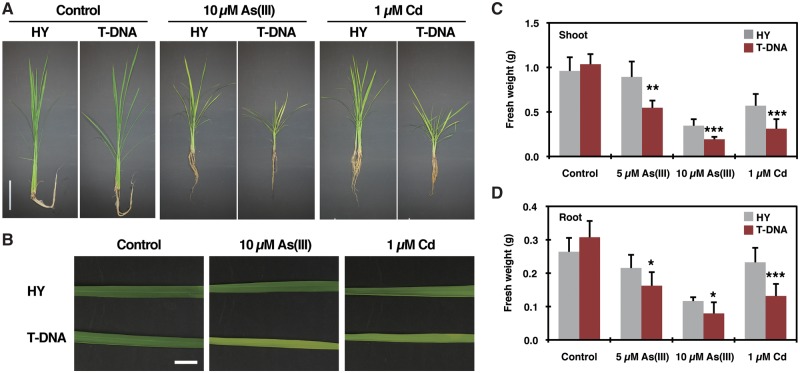
Sensitivity assay of the *OsPCS1* T-DNA insertion mutant rice under As(III) or Cd stress. (A–C) The wild-type HY and the T-DNA mutant of *OsPCS1* were hydroponically grown for 2 weeks without As(III) or Cd addition and then grown further for 3 weeks with or without As(III) or Cd. (A and B) Phenotypes of HY and the T-DNA seedlings (A) and leaf blades (B) after cultivation. Scale bar = 10 cm (A) or 1 cm (B). (C and D) Fresh weight of shoots (C) and roots (D) after cultivation. Data represent means with the SD of two independent experiments (*n* = 6–10). Asterisks indicate significant differences from the wild-type HY (*P < 0.05, **P < 0.01, ***P < 0.001; *t*-test).

As(III) and Cd tolerance of the mutants as well as the respective wild-type plants were then examined in a hydroponic culture system (Fig. 4; Supplementary Fig. S7). The T-DNA mutant exhibited increased toxicity derived from As(III) or Cd treatment compared with the wild-type HY, while the growth overall under control conditions was comparable between HY and the T-DNA line (Fig. 4A, B). These phenotypes of the T-DNA mutant were further confirmed by measuring biomass. Both shoot (Fig. 4C) and root (Fig. 4D) fresh weight were significantly lower in the T-DNA plants than in HY when exposed to As(III) (5 or 10 µM) or Cd (1 µM). Corresponding yet weaker phenotypes were observed for the second allele NG5045 (Supplementary Fig. S7). The root growth of the *Tos17* mutant was significantly more reduced by As(III) and Cd treatments compared with that of NB (Supplementary Fig. S7A, C), while the difference between the mutant and wild-type plants was not significant for the shoot (Supplementary Fig. S7B). Taken together, these results for two independent mutants demonstrate that *OsPCS1* plays a major role in achieving tolerance to As(III) and Cd stress in rice plants.

### Distribution of As and Cd is altered in shoot and grains of *OsPCS1* mutants

In addition to being essential for Cd and As tolerance, PCS has been implicated in the distribution of Cd, As and Zn in *A. thaliana* (Chen et al. 2006, Liu et al. 2010, Kühnlenz et al. 2016). We thus examined accumulation patterns of As and Cd in the *OsPCS1* mutant plants in comparison with the respective wild-type plants. First, concentrations of As and Cd in shoots and roots obtained from the hydroponic culture were measured (Supplementary Fig. S8). The As concentration in shoots was slightly yet not significantly higher both in NG5045 (*P* = 0.16, vs. NB) and in T-DNA (*P* = 0.13, vs. HY) compared with each wild-type line. The As distribution (shoot/root) was significantly higher in the T-DNA line compared with HY. In contrast to As, the Cd concentrations in shoots of the two mutants were significantly lower than in those of the wild-type plants. These results indicated the possibility that disruption of *OsPCS1* differentially affects distribution of As and Cd to shoots.

**Fig. 5:**
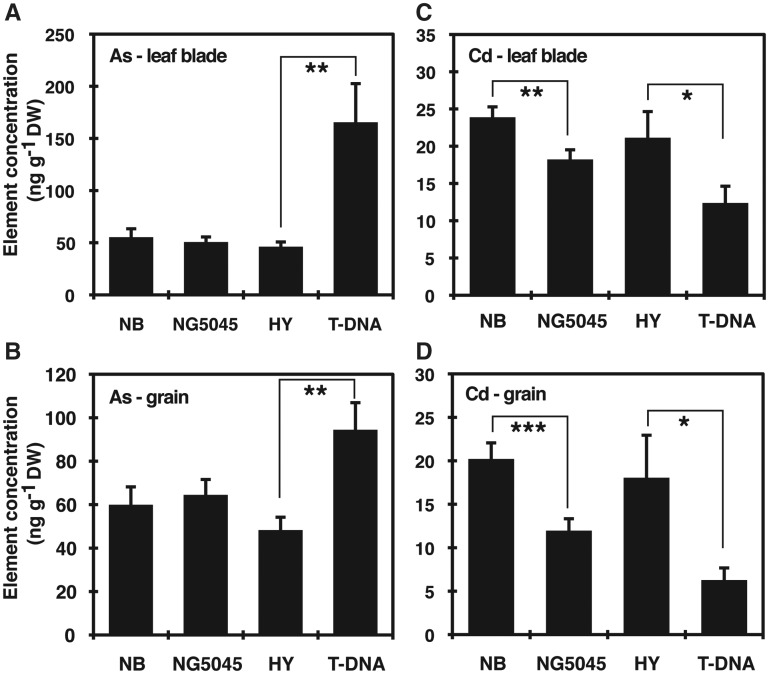
As and Cd concentrations in soil-grown *OsPCS1* mutant rice and the respective wild-type plants. The wild-type and *OsPCS1* mutant rice were grown in pots until grain ripening. After harvest, element concentrations in leaf blade and brown rice were measured by ICP-MS. Plants were grown under flooded or intermittent irrigated conditions for testing As accumulation or Cd accumulation, respectively. (A and B) As concentrations in leaf blade (A) and grain (brown rice) (B). (C and D) Cd concentrations in leaf blade (C) and grain (brown rice) (D). Data represent means with the SD of two independent experiments (*n* = 8–12). Asterisks indicate significant differences between each wild-type and mutant pair (*P < 0.05, **P < 0.01, ***P < 0.001; *t*-test).

Elemental analyses were further conducted on plant samples obtained from pot experiments. Plants were grown in a commercial nursery soil until grain ripening under flooded conditions for As determination or under intermittent irrigated conditions for Cd determination. The applied concentrations are environmentally relevant (Supplementary Table S2). The flag leaf As concentration was significantly higher in the T-DNA line than the wild-type HY, whereas no difference was found between NG5045 and NB (Fig. 5A). Similar patterns were observed for grain As concentration: As concentration in grains was nearly 2-fold higher in the T-DNA line than in HY, while no significant difference was observed between NG5045 and NB (Fig. 5B). Accumulation patterns of Cd in flag leaves and grains were also well correlated among the tested plants. The Cd concentrations in the leaves of NG5045 and T-DNA were 24% and 41% lower than in the respective wild-type plants (Fig. 5C), and the grains of NG5045 and T-DNA accumulated about 40% and 65% less Cd, respectively, compared with each wild-type line (Fig. 5D). These results suggest that disruption of OsPCS1 function has opposite effects on the accumulation of As and Cd in shoots and grains of rice plants. We further quantified As accumulation in the plants grown under intermittent irrigation conditions (Supplementary Fig. S9). Overall As concentrations in the plants were lower than those in the plants grown under flooded conditions. No significant difference was found in both flag leaf and grain As concentrations between the wild-type plants and mutant plants.

**Fig. 6:**
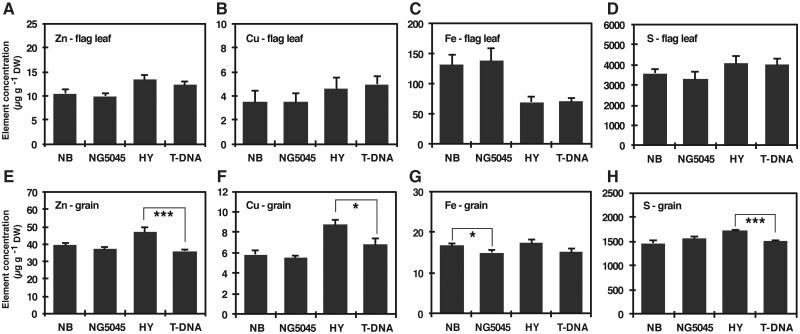
Nutrient concentrations in the soil-grown *OsPCS1* mutant rice and the respective wild-type plants. The wild-type and *OsPCS1* mutant rice were grown in pots until grain ripening under intermittent irrigated conditions. After harvest, element concentrations in leaf blade and grain (brown rice) were measured by ICP-MS. (A–D) Zn (A), Cu (B), Fe (C) and S (D) concentrations in flag leaf blade. (E–H) Zn (E), Cu (F), Fe (G) and S (H) concentrations in grain. Data represent means with the SD of two independent experiments (*n* = 8). Asterisks indicate significant differences between each wild type and mutant pair (*P < 0.05, **P < 0.01; *t*-test).

Also, we examined nutrient element concentrations in the same plant samples obtained from the intermittent irrigated cultivation (Fig. 6). In the flag leaves, there was no significant difference for Zn, Cu, Fe and S concentrations between the mutants and respective wild-type plants (Fig. 6A–D). However, the grains of the T-DNA line showed significantly reduced accumulation of Zn, Cu and S compared with HY, whereas such differences were not observed for NG5045 and NB (Fig. 6E, F, H). The grains of NG5045 showed slight but significant reduction of Fe concentration but the T-DNA line did not (Fig. 6G).

## Discussion

### Structural characteristics and expression of *OsPCS* gene variants

Like other higher plants, rice has two *PCS* genes (Kühnlenz et al. 2014). The RAP-DB and MSU database suggested three different transcript variants for *OsPCS1* (Os06g0102300 and LOC_Os06g01260 in RAP-DB and MSU, respectively, designated as *OsPCS2* in Das et al. 2017 and Hayashi et al. 2017) based on transcript evidence, and a shorter open reading frame (ORF) for *OsPCS2* (Os05g0415200/LOC_Os05g34290, designated as *OsPCS1* in Das et al. 2017 and Hayashi et al. 2017). Albeit with several differences from these database entries, recent studies isolated *OsPCS* transcript variants from IR64, an *indica* cultivar (Das et al. 2017) and the *japonica* cultivar Koshihikari (Hayashi et al. 2017). However, the genomic situation of *OsPCS* genes was not fully established. In the present study, we isolated four *OsPCS1* variants and the *OsPCS2* transcript from the model *japonica* cultivar NB basically in accordance with the database predictions and established the transcriptional and functional characteristics of the rice *PCS* genes. Our naming of the *OsPCS* genes as 1 and 2 was essentially based on the fact that Os06g0102300/LOC_Os06g01260 gives rise to a full-length PCS with higher similarity to the major PCS in *A. thaliana*, AtPCS1, then to the minor AtPCS2.

First, based on the sequence analyses, we suggest two TSSs and an alternative splicing site at exon 4/3 for *OsPCS1*, which together result in the generation of four different transcripts (Fig. 1A, B). Three of them (*OsPCS1full*, *1a* and *1b*) are identical to the database annotation, while *OsPCS1c* was not predicted. *OsPCS1full* was identical to the reported longest clone from the *indica* cultivar IR64 (accession No. KU670827) (Das et al. 2017). However, our three other *OsPCS1* clones are different from the second *indica* clone (accession No. KU670828) which has a non-spliced intron 1 (Das et al. 2017). Clearly, possible genotypic variation in splicing variants will have to be further examined in various *japonica* and *indica* cultivars.

Sequence and functional analyses suggest OsPCS1full as the rice PCS most similar to AtPCS1 with respect to PCS activity: like AtPCS1, OsPCS1full possesses both the N-terminal catalytic and the C-terminal domain implicated in metal activation of the enzyme (Fig. 2A). More importantly, its expression complemented both the *S. pombe* PCS mutant *Δpcs* (Fig. 2B–E) and *A. thaliana cad1-3* (Fig. 3) by mediating substantial PC synthesis under As(III) and Cd exposure. Other OsPCS1 variants did not show significant PCS activity, probably due to the lack of either the N- or C-terminal domain. We also examined the expression profiles of all *OsPCS* variants which we isolated (Fig. 1C). Since *OsPCS1full* showed the highest abundance among *OsPCS* transcripts in rice plants (Fig. 1C; Supplementary Fig. S1), we suggest that OsPCS1full encoded by Os06g0102300/LOC_Os06g01260 is physiologically the major form of OsPCS1.

A unique feature of OsPCS2, the secondary rice PCS according to our data, is the lack of the C-terminal domain due to an early stop codon in exon 3 of the *OsPCS2* gene (Figs. 1A, 2A; Supplementary Fig. S2). The genomic sequence around exon 3 is conserved in the *indica* cultivar Guangluai4 and the *japonica* cultivar Koshihikari according to the public short read assembly data (RAP-DB). However, recent studies reported longer *OsPCS2* transcripts from cultivars IR64 (Das et al. 2017) and Koshihikari (Hayashi et al. 2017). We therefore examined public RNA-seq data obtained from normally grown NB seedlings (Secco et al. 2013) available in the RAP-DB. We found an indication of an alternative splicing site just before the early stop codon of exon 3 which could result in longer ORFs (Supplementary Fig. S10). Our shorter *OsPCS2* variant seems rather abundant in roots, whereas the longer transcript appears dominant in shoots. Another noteworthy feature of *OsPCS2* is the expression level close to that of the most abundant *OsPCS* transcript *OsPCS1full* (Fig. 1C). In Arabidopsis, *AtPCS2* is far less expressed in seedlings compared with *AtPCS1* (Cazale and Clemens 2001). With regard to the PCS activity, OsPCS2 is the only clone besides OsPCS1full that showed PCS activity in response to Cd treatment in *S. pombe* and *cad1-3* (Figs. 2E, 3E). No activity of the predicted short form of OsPCS2 was detected in our experiments upon As(III) exposure (Figs. 2D, 3D). In contrast, PCS activity in response to As was reported for the longer OsPCS2 version not covered in our analyses (Hayashi et al. 2017). It has been suggested that different regions of the AtPCS1 C-terminal domain are required for metal-specific activation of PC synthesis (Ruotolo et al. 2004, Kühnlenz et al. 2016). The different responses of the two OsPCS2 variants are another example indicating the importance of the PCS C-terminal domain for determining metal-specific activation.

### Roles of *OsPCS1* in As(III) and Cd tolerance of rice

PCS-dependent PC synthesis has been well documented as a crucial response of Arabidopsis for detoxifying cytosolic Cd, As, Hg and Pb ions as well as Zn excess (Howden et al. 1995b, Ha et al. 1999, Tennstedt et al. 2009, Fischer et al. 2014). AtPCS1 is the major PCS in Arabidopsis since the AtPCS1 null mutant *cad1-3* displays strong hypersensitivity to these elements. While previous reports investigated seed-specific RNA interference (RNAi)-mediated knock-down of *OsPCS* genes (Das et al. 2017, Li et al. 2007) and another recent study reported a mutant for *OsPCS2* (Os05g0415200/LOC_Os05g34290), no physiological data are available for *OsPCS1* (Os06g0102300/LOC_Os06g01260), encoding the highly expressed and functional PCS variant.

We identified two independent rice mutants of *OsPCS1* (Supplementary Fig. S6) and characterized them to evaluate physiological functions of OsPCS1 with respect to As(III) and Cd tolerance as well as the within-plant mobility of As and Cd. First, the hydroponic experiments demonstrated that OsPCS1 is crucial for As and Cd tolerance of rice seedlings: disruption of *OsPCS1* significantly increased sensitivity of rice against As(III) and Cd stresses (Fig. 4; Supplementary Fig. S7). The enhanced sensitivity of the mutants was more evident in the case of As(III) treatment than in the case of Cd, demonstrating the importance of the PC-dependent pathway especially for As(III) detoxification. Our results are in line with the As hypersensitivity phenotype of *osabcc1*, a rice mutant of the vacuolar ABC transporter OsABCC1 (Song et al. 2014). OsABCC1 is crucial for removing PC–As complexes from the cytosol by vacuolar sequestration, which is the final step of the PC-dependent detoxification process as demonstrated also for Arabidopsis mutants of AtABCC1 and 2 (Song et al. 2010, Song et al. 2014).

The relatively weak phenotype of the *OsPCS1* mutants observed under Cd exposure is in contrast to the clear phenotypes under As(III) stress, and again in line with observations on *OsABCC1* mutants (Song et al. 2014). Similarly, a recent report on OsPCS2 also indicates its involvement in As tolerance but little contribution to Cd tolerance (Hayashi et al. 2017). As previously discussed (Song et al. 2014), it may be attributable to the contribution of OsHMA3, a vacuolar heavy metal ATPase. Functional OsHMA3 mediates Cd sequestration in the vacuole independent of PCs and thus is an important transporter affecting radial Cd transport in roots and Cd tolerance of rice as well (Ueno et al. 2010, Miyadate et al. 2011, Sasaki et al. 2014). There is no evidence suggesting involvement of OsHMA3 in As transport. Taken together with the *OsPCS1* mutant phenotypes reported here, it is suggested that OsPCS1-dependent Cd detoxification and OsHMA3-mediated Cd sequestration independently play an important role in Cd tolerance of rice, while the PC-dependent pathway mediated by OsPCS1 and OsABCC1 rather exclusively accounts for a major fraction of the As detoxification capacity in rice. Moreover, from the report of Hayashi et al. (2017) and our data, it appears that unlike in *A. thaliana* both *PCS* genes significantly contribute to As tolerance.

### Roles of *OsPCS1* in As(III) and Cd distribution within rice plants

In addition to the significant roles of PCS for toxic element detoxification, recent data suggest involvement of PCS in element distribution within plants (Mendoza-CÓzatl et al. 2008, Liu et al. 2010, Kühnlenz et al. 2016). In rice, physiological experiments using different cultivars confirmed PC–As complex formation in the plants grown under non-toxic As exposure conditions (Batista et al. 2014). High-resolution elemental mapping in rice grown with environmentally relevant As concentrations showed co-localization of As and S in vacuoles of nodal phloem parenchyma cells (Moore et al. 2014), suggesting thiol complexation of As in the vacuoles mediated by PCs. Supporting these observations, *OsABCC1* mutant analyses suggested OsABCC1-mediated control of long-distance As transport (Song et al. 2014). Co-localization of Cd and S was also reported in rice grown in the presence of environmentally relevant Cd concentrations (Yamaguchi et al. 2012), but functions of PCS related to long-distance Cd transport toward grains had remained elusive.

In this study, using rice mutants of *OsPCS1,* we examined whether PCS affected the distribution of As and Cd into shoots and grains. The elemental analyses on the hydroponically grown plant samples first indicated contrasting effects of *OsPCS1* disruption on As and Cd accumulation in rice shoots: increased As and decreased Cd content of the mutant shoot (Supplementary Fig. S8). This suggested the possibility that OsPCS1-dependent PC synthesis confers As retention in roots but Cd translocation from roots to shoots. The independent soil experiments performed at environmentally relevant concentrations further supported the contrasting effects of *OsPCS1* disruption. An increase in As accumulation was evident in grains and leaves of the T-DNA line and a decrease of Cd accumulation in grains and leaves was significant for the two tested mutant lines (Fig. 5). The weak allele NG5045 did not show the As accumulation phenotype, unlike the T-DNA line, but this may be attributable to residual *OsPCS1* expression in NG5045 and/or to the secondary OsPCS (Hayashi et al. 2017). The OsPCS1-dependent changes in As and Cd accumulation indicated the physiological significance of OsPCS1 and roles of metal(loid)–PC complexes in the within-plant mobility of these elements, albeit in opposite directions. It should also be noted that the T-DNA line showed reduced accumulation of Zn, Cu and S in the grains (Fig. 6), indicating the possibility that *OsPCS1* is also influencing the within-plant mobility of these essential elements.

A recent study on the *OsPCS2* mutant showed the increased As accumulation in grains, but no phenotype for Cd was observed (Hayashi et al. 2017). These and our data combined suggest that in fact both *OsPCS* genes significantly contribute to restricting As mobility within rice plants. This is different from *A. thaliana* where AtPCS1 is the only PCS isoform controlling metal tolerance and accumulation. A physiological function for AtPCS2 has yet to be found.

In contrast, apparently only OsPCS1, the isoform analyzed in this study, exerts a measurable influence on Cd accumulation. The divergent effects of *OsPCS1* disruption on As and Cd accumulation in shoot may be explained again by the relatively large contribution of OsABCC1 to vacuolar transport of As and a comparatively smaller contribution for Cd. The PCS- and OsABCC1-dependent pathway largely contributes to vacuolar sequestration of As in the PC–As complex form in root cells as well as nodal phloem companion cells, resulting in As retention within cells (Song et al. 2014). Lack of *OsPCS1* would reduce formation of the PC–As complex, and thereby very probably eliminate the contribution of OsABCC1-mediated vacuolar sequestration of As. This would eventually diminish the As retention capacity of cells and thus enhance As mobility within plants. This hypothesis is basically in line with the one suggested for *OsABCC1* mutant phenotypes (Song et al. 2014), and reasonably explains the increased As accumulation in grains of both *OsABCC1* and *OsPCS1* mutants.

Regarding Cd, OsABCC1 could play a role in the vacuolar sequestration of PC–Cd complexes. However, according to yeast expression experiments, the affinity of OsABCC1 is lower for PC–Cd than for PC–As (Song et al. 2014). Thus unlike the case of As, substantial PC–Cd complex formed in wild-type root cells may not be sequestered in vacuoles, and such escaped Cd would be channeled into long-distance transport pathways. Reduced PCS activity would consequently result in lower mobility of Cd and less accumulation in above-ground tissues, as we observed for leaves and grains. It is also possible that reduced PCS activity in roots diminishes Cd uptake by roots, suggested by reduced Cd concentration in the T-DNA mutant roots (Supplementary Fig. S8D). Formation of PC–Cd complexes in the cytosol may drive Cd uptake into root cells.

An alternative explanation for the contrasting effects on Cd and As accumulation in grains could be that a reduction in As–PC complex concentrations enhances the formation of other, more mobile As complexes, for example with GSH. Such complexes have been detected in plants (Raab et al. 2005). However, we are not aware of data suggesting such higher mobility of As complexes with thiols other than PCs. It will certainly be important to address directly in future studies changes in the speciation of As and Cd caused by a loss of PCS activity.

### Potential of PCS for mitigating risks of As and Cd background contamination

Daily consumption of plant-derived foods, especially rice containing trace yet relevant levels of As or Cd, has increasingly been suggested as a potential risk to human health (Tsukahara et al. 2003, Meharg et al. 2009, Gilbert-Diamond et al. 2011, Meharg et al. 2013). For mitigating As and Cd contamination of rice grains, water management control of paddies is a possible approach immediately available in fields. However, there is a clear trade-off relationship between phytoavailability of As and Cd in soil (Arao et al. 2009, Honma et al. 2016): aerobic upland conditions drastically decrease As but increase Cd in soil solution, and, in contrast, flooded reducing conditions have completely opposite effects. Changes in major As and Cd transporters is suggested as another promising approach for reducing toxic element accumulation in grains (Uraguchi and Fujiwara 2013, Clemens and Ma 2016). However, As and Cd do not share the same uptake and distribution pathways (Clemens and Ma 2016) so that it is difficult to establish plants with both low-As and low-Cd phenotypes by manipulating a single transporter gene. Moreover, it is suggested that disruption of such transporter genes harbors a risk of disturbing nutritional homeostasis and impairing plant growth and stress resistance (Ma et al. 2006, Sasaki et al. 2012).

In this study, we establish the foundation for utilization of PCS in controlling both As and Cd partitioning into rice grains. Under environmentally relevant soil conditions, we found significantly decreased Cd accumulation in grains when *OsPCS1* is disrupted (Fig. 5). For As, although the *OsPCS1* T-DNA insertion line accumulated double the level of As in grains, NG5045, the *Tos17* mutant with residual *OsPCS1* expression, did not show such increased As accumulation. Taking advantage of the reduced Cd and unaltered As phenotypes of NG5045, it might be possible to achieve mitigation of both Cd- and As-related risks by cultivating NG5045 in fields with rather aerobic conditions which diminish the phytoavailable As fraction in soil solutions. Indeed, our pot experiment with intermittent water management suggests the practical potential of the idea: the *Tos17* mutant line showed the reduced grain Cd accumulation attributable to *OsPCS1* disruption, and the As levels in NG5045 as well as NB were reduced to <20% of the flooded conditions (Supplementary Fig. S9) due to the relatively aerobic soil condition. It should be noted that NG5045 showed wild-type-like vegetative growth under control conditions (Supplementary Fig. S7) and little alteration of nutrient accumulation (Fig. 6). Performance of the non-transgenic *OsPCS1* allele NG5045 especially in regard to As and Cd accumulation as well as yields should be further examined in field conditions. More generally, it appears promising to explore systematically the effects of different *PCS* alleles on toxic metal accumulation in cereal grains.

## Materials and Methods

### Plant materials and growth conditions

Rice (*Oryza sativa* L.) cv. NB was used for the cloning of *OsPCS* variants and expression analyses. A T-DNA insertion line (PFG_2D-20992) and *Tos17* insertion lines (NG5039, NG5045 and NG5071) were obtained from the Rice T-DNA Insertion Sequence Database Center at Pohang University of Science and Technology (POSTECH) and from the Rice *Tos17* Insertion Mutant Database at the National Institute of Agrobiological Sciences (NIAS), respectively. Homozygous plants for each line were selected by PCR with specific primers (Supplementary Table S1) and then used for the characterization. NB and HY were used as the wild types of *Tos17* and the T-DNA line, respectively.

For hydroponic culture of rice, half-strength Kimura B solution supplemented with 2 mM MES (pH 5.6, KOH) was used (Uraguchi and Fujiwara 2011) and plants were grown under long-day conditions (16 h light/8 h dark, 26°C/23°C). Soil experiments were conducted in a temperature-controlled greenhouse (25–30°C with natural light conditions). A commercial nursery soil (‘Honens nursery soil No. 1’, Honen Agri Co.) containing basal fertilizers was used for the pot experiment.

For Arabidopsis complementation, *A. thaliana* wild-type Col-0 and the AtPCS1 null mutant *cad1-3* were used. Agar plates containing one-tenth modified Hoagland medium (Tennstedt et al. 2009, Fischer et al. 2014) were used for cultivation of Arabidopsis plants [100 µM (NH_4_)_2_HPO_4_, 200 µM MgSO_4_, 280 µM Ca(NO_3_)_2_, 600 µM KNO_3_, 5 µM Fe-HBED, 1% (w/v) sucrose, 5 mM MES, 1% (w/v) agar, pH 5.7]. Purified agar (Nacalai Tesque) was used for Cd tolerance assay, and Type E agar (Sigma-Aldrich) was used for other experiments. For PC analyses, the following microelements were added to the medium: 4.63 µM H_3_BO_3_, 32 nM CuSO_4_, 915 nM MnCl_2_, 77 nM ZnSO_4_ and 11 nM MoO_3_ (Kühnlenz et al. 2014). Arabidopsis seeds were surface sterilized and sown on agar plates. After 2 d stratification at 4°C, plants were grown vertically in a growth chamber (16 h light/8 h dark, 22°C).

### Isolation of *OsPCS* cDNAs

Total RNA was isolated from NB seedlings using Trizol (Thermo Fisher Scientific). Extracted RNA was reverse-transcribed using a SuperScript First-Strand Synthesis System (Invitrogen) with the oligod(T) primer and random hexamers. The synthesized cDNA was used as a template for isolation of *OsPCS* cDNAs by PCR using primers designed based on the *OsPCS* sequences in the databases (Supplementary Table S1). The amplified products were subcloned into the pGEM-T vector (Promega) and sequenced.

### Generation of *OsPCS*-expressing Arabidopsis

A 2,075 bp fragment upstream of the *AtPCS1* start codon was amplified from Col-0 genomic DNA with the primers listed in Supplementary Table S1. The obtained fragment spanning the putative *AtPCS1* promoter region was ligated into the *Hin*dIII and *Kpn*I sites of pTS100 (Uraguchi et al. 2011), which has *sGFP* within the att cassette of pMDC32 (Curtis and Grossniklaus 2003). The resulting plasmid was named pSUB59. The coding sequences of *OsPCS1full*, *OsPCS1a*, *OsPCS1b* and *OsPCS2* were amplified from the pGEM-T vectors harboring each cDNA, and *sGFP* was amplified from pTS100 using the primers listed in Supplementary Table S1. The respective amplicons were ligated into the *Kpn*I and *Pac*I sites of pSUB59 to obtain pSUB60 (*ProAtPCS1-OsPCS1full*), pSUB61 (*ProAtPCS1-OsPCS1a*), pSUB62 (*ProAtPCS1-OsPCS1b*), pSUB64 (*ProAtPCS1-OsPCS2*) and pSUB65 (*ProAtPCS1-sGFP*). The plasmids were introduced into *Agrobacterium tumefaciens* GV3101::pMP90, which were then used for transformation of *cad1-3* plants by the floral dip method (Clough and Bent 1998). Transformants were selected on agar medium containing hygromycin, and T_3_ homozygous lines were used for the experiments.

### Expression analyses

Rice seedlings hydroponically grown for 3 weeks were used for expression analyses of *OsPCS* genes in the wild-type and mutant plants. To examine responses to metal treatments, 3-week-old NB seedlings were transferred to a hydroponic solution containing 10 µM Cd, As(III) or Zn and treated for 3 h. Total RNA was extracted using Trizol (Thermo Fisher Scientific) from rice seedlings, followed by DNase I treatment (Thermo Fisher Scientific). Obtained RNA was then used for cDNA synthesis by PrimeScript RT Master Mix (TAKARA). Quantitative real-time PCR was performed with iQ SYBR Green Supermix (BioRad). Semi-quantitative RT–PCR was conducted with PrimeSTAR HS DNA Polymerase (TAKARA). *OsUBQ5* served as an internal control (Uraguchi et al. 2011). For semi-quantitative RT–PCR, the relative intensity of the obtained bands was quantified by Image J software.

To examine introduced *OsPCS* expression in the Arabidopsis transformants, Arabidopsis plants grown on Hoagland agar plates for 12 d were subjected to RNA extraction. An RNeasy Plant Mini (QIAGEN) was used for total RNA extraction from the seedlings, and DNase (QIAGEN) treatment was applied during extraction. PrimeScript RT Master Mix (TAKARA) was used for cDNA synthesis, and RT–PCR was performed with GoTaq Green Master Mix (Promega). *Elongation factor 1α* (At5g60390) served as an internal control. The primer sequences used for expression analyses are listed in Supplementary Table S2.

### Heterologous expression of OsPCS variants in *Schizosaccharomyces pombe*

The *S. pombe PCS* knockout strain *Δpcs* (Clemens et al. 1999) heterologously expressing OsPCS variants was used for assaying PCS activity. The *OsPCS* coding sequences without a stop codon were amplified from the pGEM-T vectors harboring each *OsPCS* cDNA and respectively ligated into the *Xho*I and *Not*I sites of pSGP72, a fission yeast expression vector. The resulting plasmids were used for transformation of *Δpcs.* Cells carrying the empty vector served as negative control. Cells carrying pSGP72 with *AtPCS1* or *AtPCS2* (Kühnlenz et al. 2014) were also included in the assay.

Yeast cultivation was carried out as described previously with some modifications (Kühnlenz et al. 2016). Yeast cultivation was carried out at 30°C in Edinburgh minimal medium (EMM). Pre-cultured cells were inoculated to an OD_600_ = 0.1 in EMM supplemented with 20 mM thiamine and grown overnight. Then cells were washed twice in EMM supplemented with 1 µM thiamine and inoculated at an OD_600_ = 0.1 in EMM supplemented with 1 µM thiamine in the presence or absence of either Cd or As(III). For growth assay, three different concentrations of As(III) (0.2, 0.4 and 1 mM) and of Cd (1, 2 and 5 µM) were applied and growth of the cells was monitored by measuring OD_600_ for 24 h. For PC analysis, 10 µM As(III) or 10 µM Cd was applied. After 24 h incubation, cells were harvested, frozen in liquid N_2_ and lyophilized for PC extraction and analysis.

### Phenotyping of the *OsPCS*-expressing transgenic Arabidopsis lines

For metal tolerance assays, Arabidopsis plants were grown on the agar plates containing Cd (2 or 3 µM CdCl_2_) or As(III) (1 or 1.5 µM NaAsO_2_) for 12 d. The plates without supplementation of Cd or As(III) served as controls. Plant growth was assessed by root length measurement at the end of the cultivation.

For PC analyses, Arabidopsis plants were grown on the control plates for 7 d. Uniformly grown seedlings were then transferred to plates containing 5 µM As(III) or Cd and incubated for an additional 7 d. Roots were then separated from shoots and were frozen in liquid nitrogen after fresh weight measurement. Homogenously ground materials were used for further PC analysis as described below.

### Phenotyping of *OsPCS1* mutant rice

For PC analyses, rice plants were grown hydroponically with half-strength Kimura B medium for 2 weeks. Established seedlings were then transferred to the medium supplemented with 10 µM Cd. Roots were harvested 3 d after transfer and were frozen in liquid nitrogen after fresh weight measurement. Homogenously ground material was used for further PC analysis as described below.

Hydroponic experiments were carried out to examine Cd and As(III) sensitivity and element accumulation of *OsPCS1* mutant rice. Two-week-old seedlings grown with half-strength Kimura B medium were exposed to the medium supplemented with 1 µM CdCl_2_, or 5 or 10 µM NaAsO_2_ for 3 weeks. The medium without Cd or As(III) addition served as control. The hydroponic solution was renewed every week. Fresh weights of roots and shoots were separately measured at harvest, and roots were subjected to sequential washing procedures: roots were desorbed for 10 min each in ice-cold MilliQ water, 20 mM CaCl_2_ (twice), 10 mM EDTA (pH 5.7) and MilliQ water. Harvested roots and shoots were dried at 50°C before elemental analysis.

Soil experiments were conducted to investigate elemental profiles of *OsPCS1* mutant rice grown in soil containing environmentally relevant levels of Cd and As (Supplementary Table S2). Three-week-old seedlings grown in a nursery box were transferred to 1/5,000 a Wagner pots filled with 2.3 kg of the soil. The mutants were paired with each wild-type plant in a pot and grown until the grain ripening stage. Six pots were set in a plastic container and three or four containers were prepared for each irrigation condition. Plants for determining As accumulation were grown under flooded conditions, whereas intermittent water irrigation was applied for plants subjected to Cd and As determination. Water levels in plastic containers for the flooded condition were maintained to cover the soil surface with 2–3 cm depth. For the intermittent irrigated condition, when the water level was lowered close to the bottom of the plastic containers, plants were watered to the level covering the soil surface (once or twice a week). Flag leaves and panicles were harvested and dried at 50°C before elemental analysis. Dehusked grains (brown rice) were used for acid digestion.

### Phytochelatin analysis

All plant material was frozen in liquid nitrogen and ground to a homogenous powder. *Schizosaccharomyces pombe* samples were frozen in liquid nitrogen and lyophilized. Thiols were extracted and derivatized as described (Kühnlenz et al. 2014). Thiol derivatives were analyzed by HPLC equipped with a fluorescence detector (Kühnlenz et al. 2014, Nishida et al. 2016).

### Elemental analysis

Dried plant samples (flag leaves and brown rice) were wet-digested with a mixture of HNO_3_ and H_2_O_2_ as described (Uraguchi et al. 2009, Kühnlenz et al. 2016). Inductively-coupled plasma-optical emission spectrometry (ICP-OES; iCAP 6500, Thermo Fisher Scientific) and inductively-coupled plasma-masss spectrometry (ICP-MS; Agilent 7800, Agilent Technologies) were used for elemental quantification of samples from hydroponic experiments and soil experiments, respectively.

To determine extractable element concentrations in the soil, 1 M HCl extraction was conducted for As determination (Kuramata et al. 2013), and 0.1 M HCl extraction was applied for determination of Cd and other elements (Uraguchi et al. 2009), according to the Agricultural Land-Soil Pollution Prevention Act in Japan. ICP-OES (iCAP7400Duo, Thermo Fisher Scientific) was used for element quantification in soil extract samples.

## Supplementary data

Supplementary data are available at PCP online.

## Funding

This work was supported by the Japan Society for the Promotion of Science [grant Nos. 15H06580 and 16K14873 to S.U.] and the Deutsche Forschungsgemeinschaft [CL 152/7-1 and CL 152/7-2 to S.C.].

## Supplementary Material

Supplementary Figures and Tables

## Abbreviations

EMM: Edinburgh minimal medium
GSH: glutathione
HY: Hwayoung
ICP-MS: inductively-coupled plasma-mass spectrometry
ICP-OES: inductively-coupled plasma-optical emission spectrometry
MA: mugineic acid
NA: nicotianamine
NB: Nipponbare
ORF: open reading frame
PC: phytochelatin
PCS: phytochelatin synthase
RT–PCR: real-time reverse transcription–PCR
TSS: transcription start site
