# Supplementary material for: Phytochelatin Synthase has Contrasting Effects on Cadmium and Arsenic Accumulation in Rice Grains
Source: Plant Cell Physiol. 2017 Aug 14;58(10):1730–42. doi: 10.1093/pcp/pcx114 (PMC5914395; doi:10.1093/pcp/pcx114)
Supplement: Supplementary Figures and Tables [file pcp-2017-e-00286-file008_pcx114.pdf]

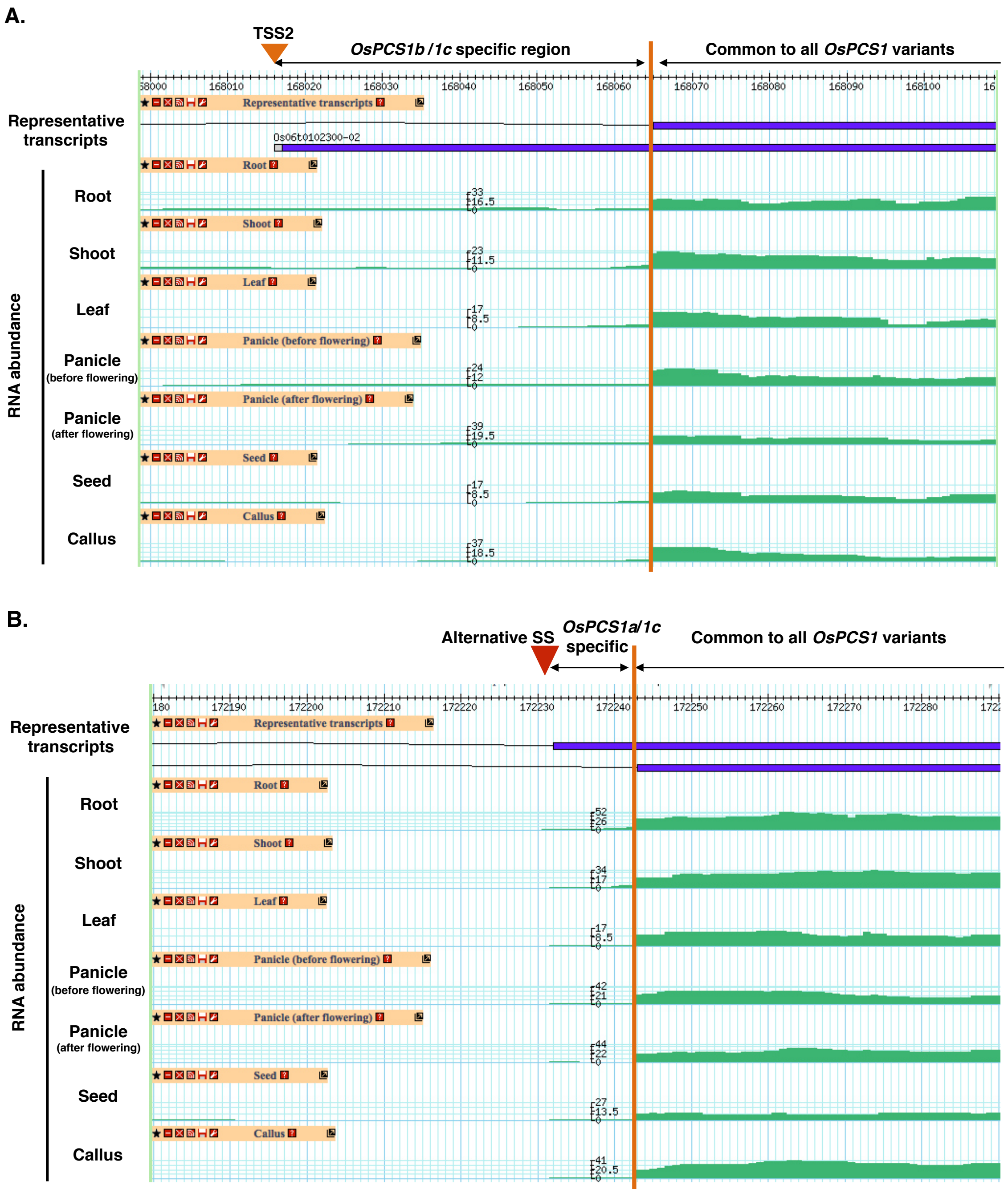

Supplementary Figure S1. Genomic and transcriptional situation of *OsPCS1* locus (Os06g0102300/LOC_Os06g01260). Original images were captured from the RAP-DB database showing transcript abundance of *OsPCS1* in various rice tissues and callus. A, Transcript abundance in the region starting from TSS2 (predicted secondary transcript starting site specific to *OsPCS1b/1c,* Fig. 1A) and the flanking *OsPCS1*s common region separated by an orange line. In all tested tissues, the expression level is much lower in the

OsPCS1b/1c specific region than in the *OsPCS1* common region, suggesting higher expression of *OsPCS1full* and/or *OsPCS1a*. B, Transcript abundance in the 11 bp-region after the alternative splicing site (SS) specific to *OsPCS1a/1c* (Fig. 1A) and the flanking *OsPCS1*s common region separated by an orange line. The expression level is much lower in the *OsPCS1a/1c* specific region than in the *OsPCS1* common region. Taken together, these results suggest that *OsPCS1full* is a major variant with higher expression levels in various rice tissues.

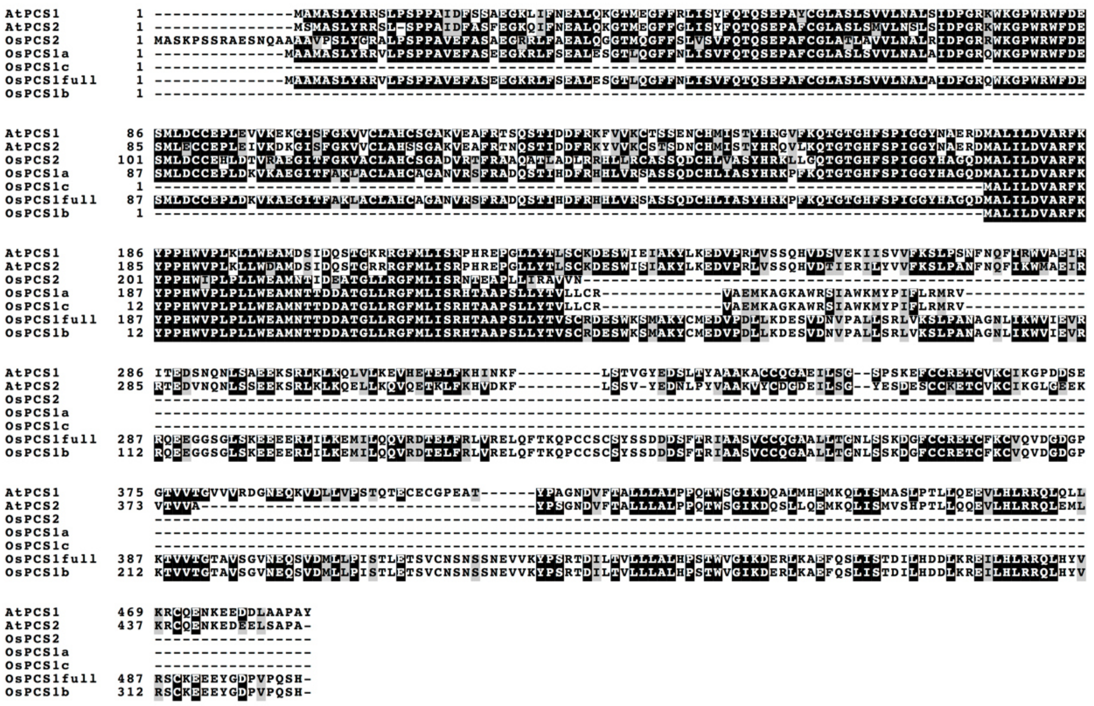

Supplementary Figure S2. Alignment of rice and Arabidopsis PCS amino acid sequences.

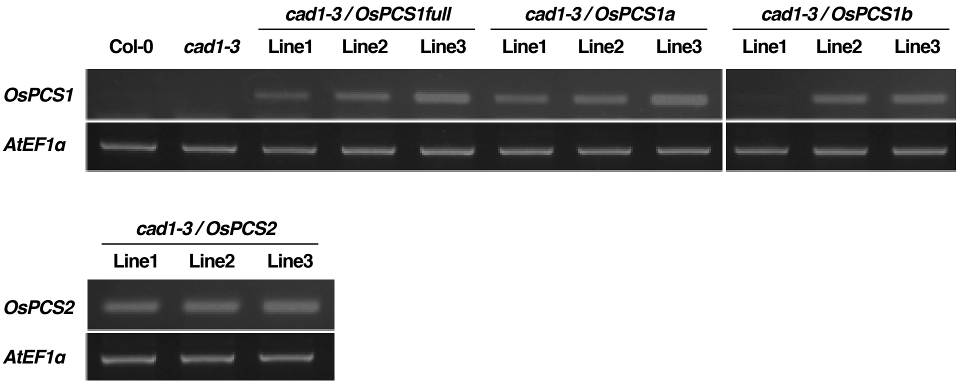

Supplementary Figure S3. Expression analyses of *OsPCS* transgenes in the Arabidopsis transgenic plants by RT-PCR.

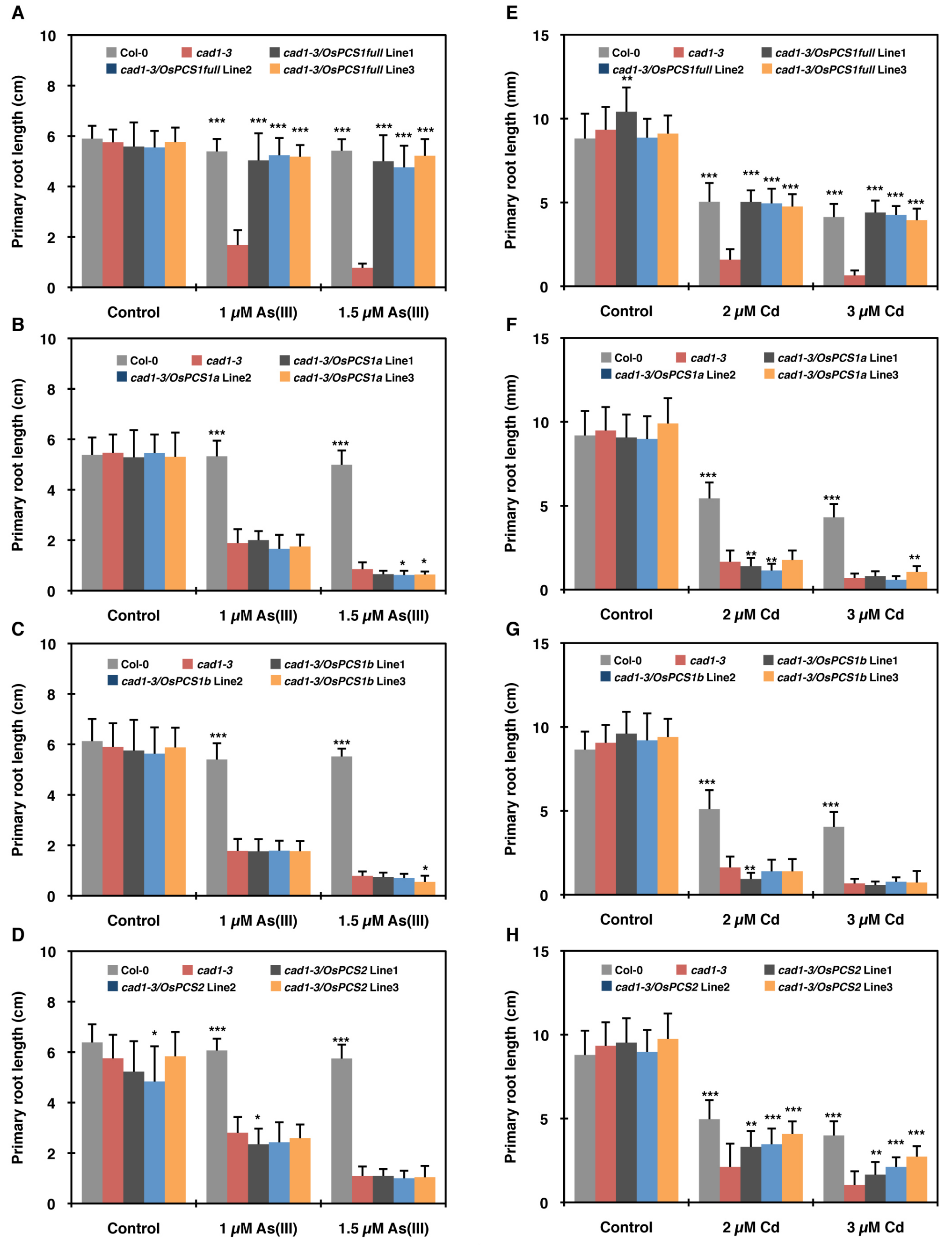

Supplementary Figure S4. As and Cd sensitivity assay of the Arabidopsis plants harboring *OsPCS* transgenes. Primary root lengths were measured after 12 d cultivation on the agar plates containing As(III) (A to D) or Cd (E to H). A and E, *cad1-3/OsPCS1full*. B and F, *cad1-3/OsPCS1a*. C and G, *cad1-3/OsPCS1b*. D and H, *cad1-3/OsPCS2*. Data represent means with SD of two independent experiments (*n* = 13 - 25). Asterisks indicate significant differences from *cad1-3* within each treatment (* p<0.05, ** p<0.01, *** p<0.001; *t*-test).

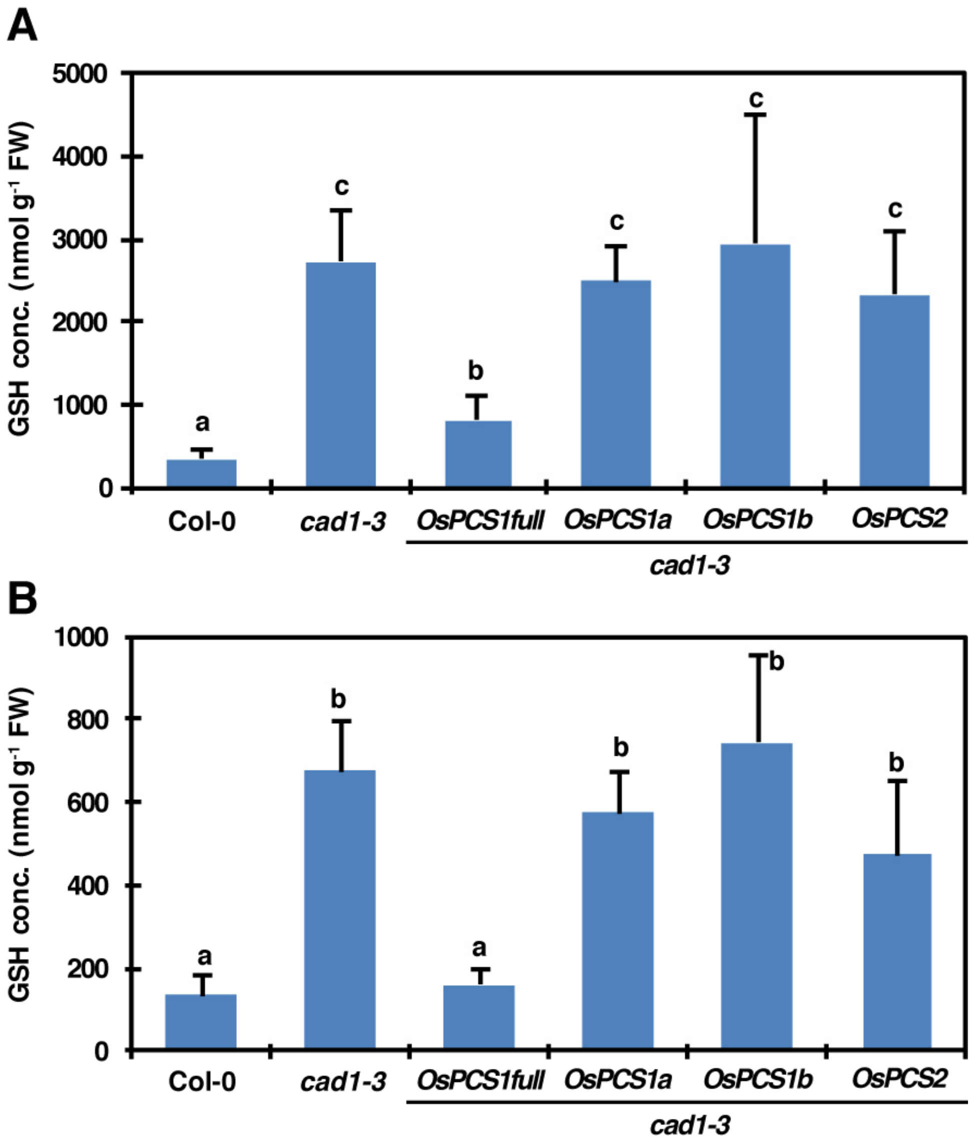

Supplementary Figure S5. GSH concentrations in roots of Col-0, *cad1-3* and *cad1-3* transgenic plants with *OsPCS* exposed to As(III) (A) or Cd (B). 7 d old seedlings were transferred from the control medium to the medium containing 5µM As(III) or 5µM Cd and grown for 7 d before harvest. Data represent means with SD of two independent experiments (n = 3 - 4). Means sharing the same lowercase (PC2) and uppercase (PC3) letter are not significantly different (p<0.05, Tukey's HSD).

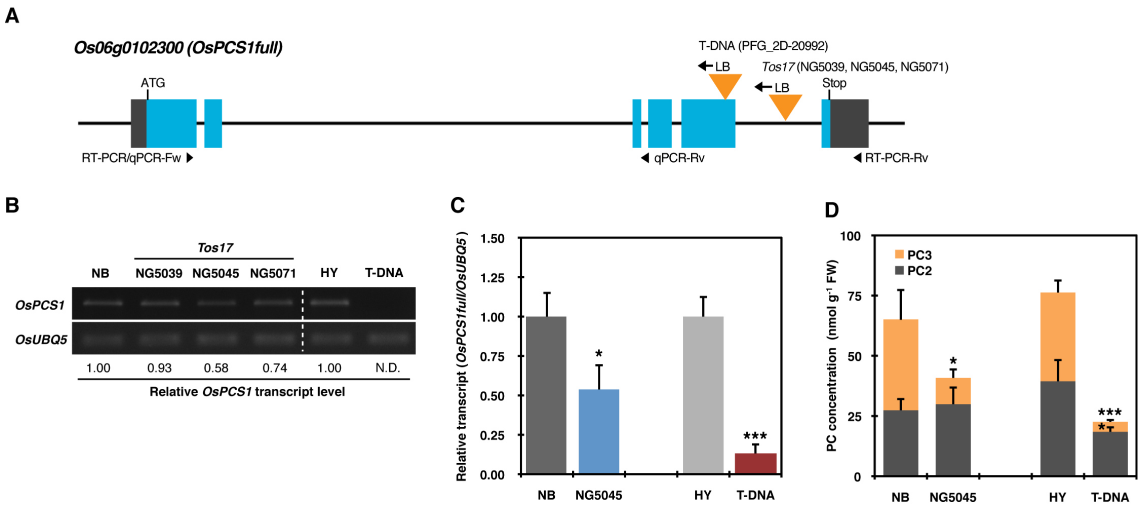

Supplementary Figure S6. Characterization of *OsPCS1* mutant rice. A, Insertion sites of T-DNA and *Tos17* in Os06g0102300 (*OsPCS1full*). Black and blue boxes represent untranslated regions and coding regions, respectively. B and C, *OsPCS1* expression analyses in the wild-type and mutant plants by RT-PCR (B) and quantitative real-time PCR (C). D, PCs concentrations in the rice roots exposed to 10μM Cd for 3 d. C and D, Data represent means with SD of at least three biological replicates. Asterisks indicate significant differences from each wild-type line (* $p<0.05$, ** $p<0.01$, *** $p<0.001$; *t*-test).

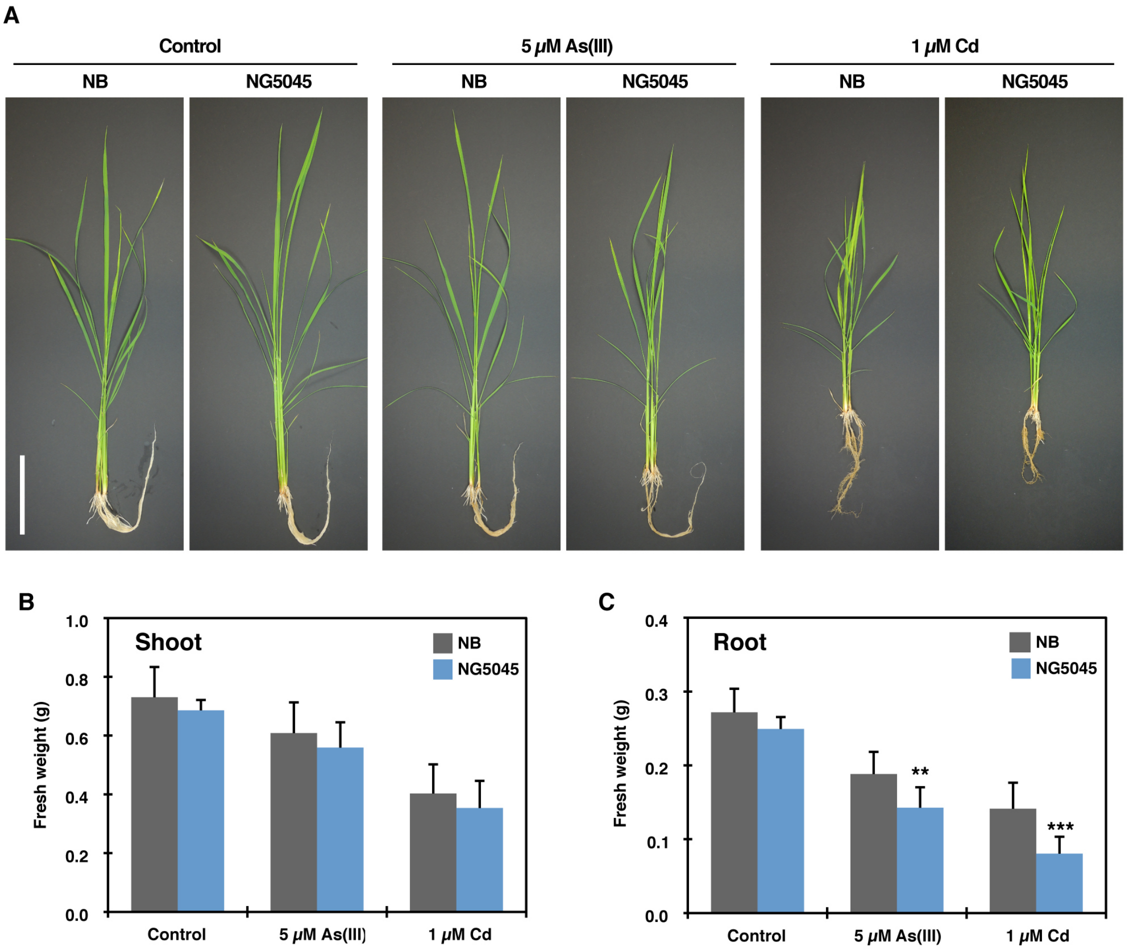

Supplementary Figure S7. As and Cd sensitivity assay of the *Tos17* insertion rice. A to C, the wild-type NB and *Tos17* mutant of *OsPCS1* (NG5045) were hydroponically grown for 2 weeks without As(III) or Cd addition and then grown further for 3 weeks with or without 5 μM As(III) or 1 μM Cd. A, Phenotypes of the NB and NG5045 after cultivation. A white bar = 10cm. B and C, Fresh weight of shoot (B) and root (C) after cultivation. Data represent means with SD of two independent experiments (n = 6 - 10). Asterisks indicate significant differences from the wild-type NB (** p<0.01, *** p<0.001; t-test).

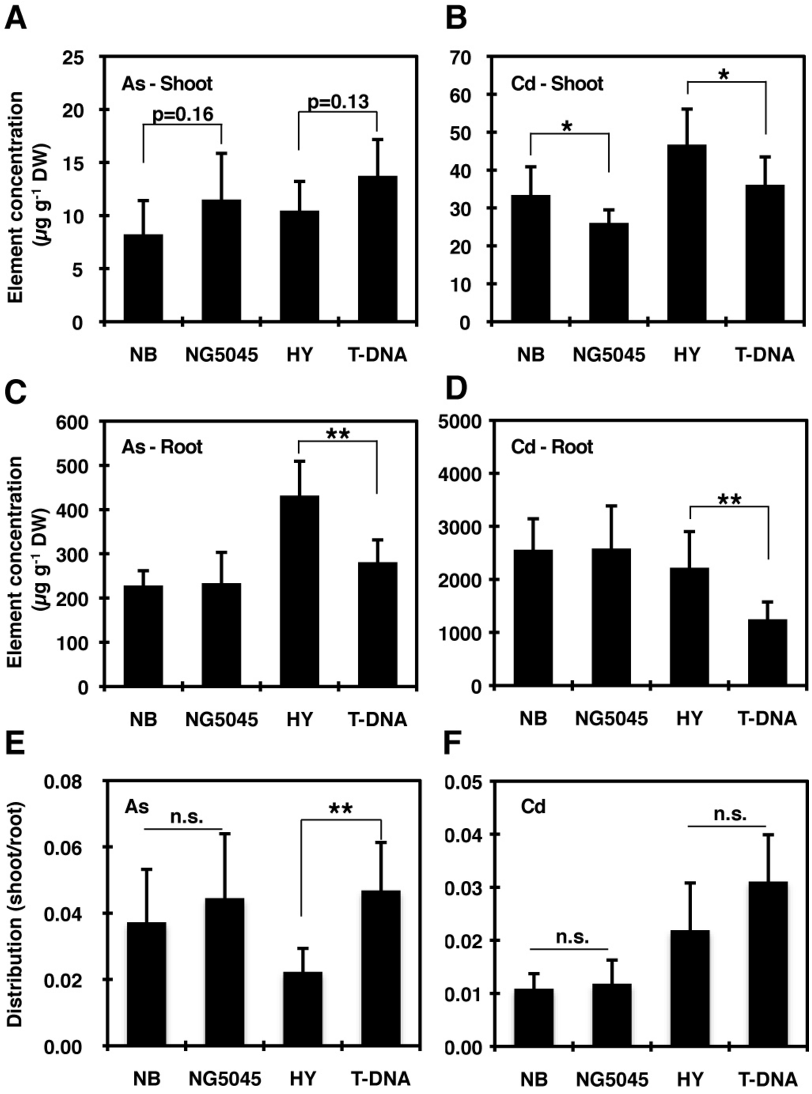

Supplementary Figure S8. As and Cd accumulation in the hydroponically grown *OsPCS1* mutant rice and the respective wild-type plants. A to F, The wild-type and *OsPCS1* mutant rice were hydroponically grown for 2 weeks without As(III) or Cd addition and then grown further for 3 weeks with or without 5 µM As(III) or 1 µM Cd. A, As concentration in shoot. B, Cd concentration in shoot. C, As concentration in root. D, Cd concentration in root. E, As distribution ratio. E, Cd distribution ratio. Data represent means with SD of two independent experiments (n = 6 - 8). Asterisks indicate significant differences between each wild-type and mutant pair (* p<0.05, ** p<0.01; t-test).

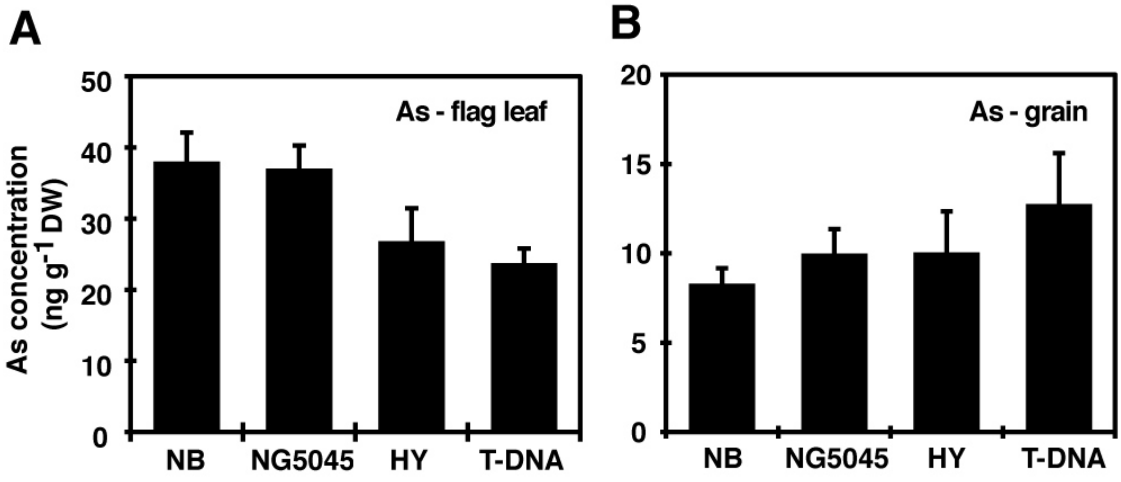

Supplemental Figure S9. As concentrations in *OsPCS1* mutant rice and the respective wild-type plants grown under intermittent water conditions. The wild-type and *OsPCS1* mutant rice were grown in pots until grain ripening. After harvest, element concentrations in leaf blade and brown rice were measured by ICP-MS. A and B, As concentrations in flag leaf blade (A) and grain (B). Data represent means with SD of two independent experiments (n = 11 - 12).

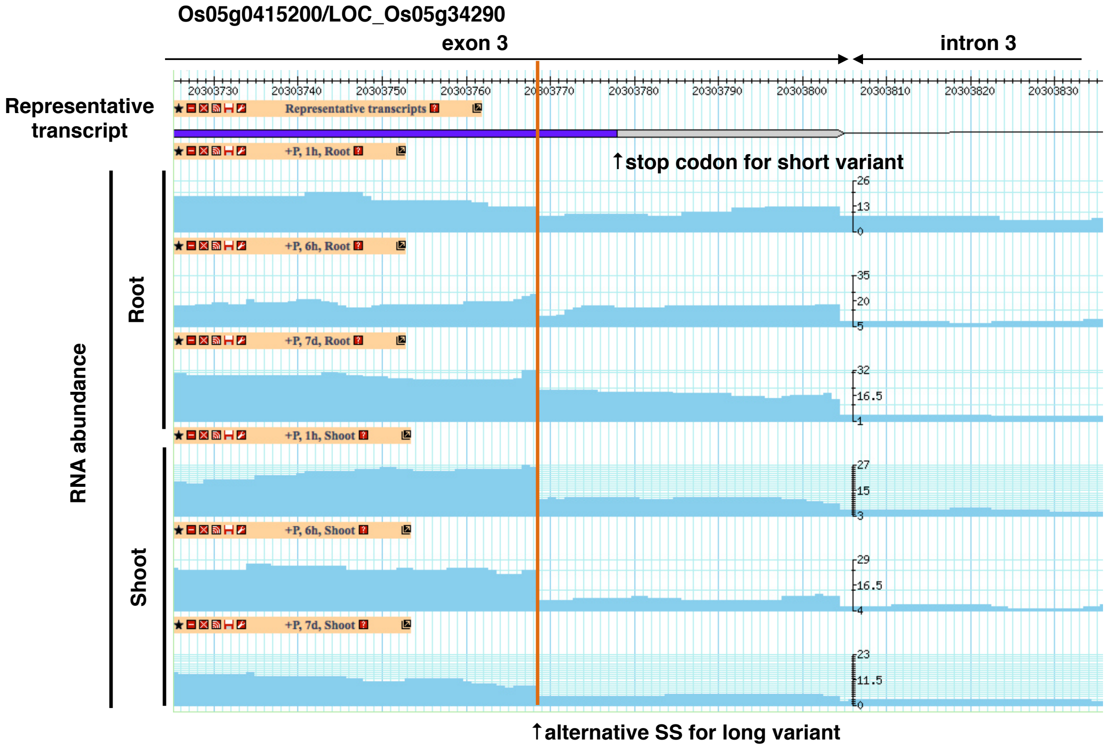

Supplementary Figure S10. Genomic and transcriptional situation of *OsPCS2* locus (Os05g0415200/LOC_Os05g34290). Original image was captured from the RAP-DB database showing transcript abundance of *OsPCS2* in roots and shoots. Decrease of transcript abundance after a possible alternative splicing site indicated with an orange line is much evident in shoot, suggesting the long variant is dominant in shoot. In root such remarkable decrease is not observed, suggesting the short variant is dominant in root.

Supplemental Table S1 List of primers used in the study

Primer set for	Primer name	Primer sequence (5' to 3')
Cloning of *OsPCS1* cDNA	OsPCS1full/a 5'UTR Fwd	TCGAGTGCAAGAAGAGGAAAGC
	OsPCS1b/c 5'UTR Fwd	CATTGCATTGCATTTCAGAC
	OsPCS1 3'UTR Rev	CTTTTGCAAGGACACCAGCC
Cloning of *OsPCS2* cDNA	OsPCS2 Fwd	ATGGCGTCTAAACCAAGCAG
	OsPCS2 3'UTR Rev	CCAGCATTGGGAGGAAGATG
Plasmid construction for Arabidopsis transforma	ProAtPCS1 HindIII Fwd	ccCAAGCTTCTCATGTTTATTGAAC
	ProAtPCS1 KpnI Rev	ggggtaccTTTTCACTGCTTGTTTTGGT
	OsPCS1full/a KpnI Fwd	ggggtaccATGGCAGCGATGGCATCCCT
	OsPCS1full/b PacI Rev	ccttaattaaTTAATGGGATTGTGGCACAG
	OsPCS1b/c KpnI Fwd	ggggtaccATGGCGCTTATCCTGGATGTC
	OsPCS1a/c PacI Rev	ccttaattaaCTACACTCTCATCCTTAAGAAG
	sGFP KpnI Fwd	ggggtaccATGGTGAGCAAGGGCGAGG
	sGFP PacI Rev	ccttaattaaTTACTTGTACAGCTCGTCCATGCC
Plasmid construction for yeast transformation	OsPCS1full/a XhoI Fwd	ccgctcgagATGGCAGCGATGGCATCCCT
	OsPCS1b/c XhoI Fwd	ccgctcgagATGGCGCTTATCCTGGATGT
	OsPCS2 Xho1 Fwd	ccgctcgagATGGCGTCTAAACCAAGCAG
	OsPCS1full NotI Rev	ataagaatgcggccgccATGGGATTGTGGCACAG
	OsPCS1a NotI Rev	ataagaatgcggccgccCACTCTCATCCTTAAGAAGATCG
	OsPCS1b NotI Rev	ataagaatgcggccgccATGGGATTGTGGCACAGGAT
	OsPCS1c NotI Rev	ataagaatgcggccgccCACTCTCATCCTTAAGAAGATC
	OsPCS2 NotI Rev	ataagaatgcggccgccATTTACCACTGCACGGATC
RT-PCR of Arabidopsis transgenic plants	OsPCS1 common Fwd	TTCAAATACCCTCCTCACTGG
	OsPCS1 common Rev	CACTGTGTAGAGCAATGAAGGAG
	OsPCS2 Fwd	TCGCTTCAAATACCCTCCTC
	OsPCS2 Rev	TGAGATAAGCATGAACCCCC
	AtEF1a Fwd	AGCACGCTCTTCTTGCTTTC
	AtEF1a Rev	AACGCCTGTCAATCTTGGTC
Genotyping of Tos17 mutant rice	Tos17 OsPCS1 Fwd	TGCTCCCTACTCCCTAGCAA
	Tos17 OsPCS1 Rev	TGTGGCACAGGATCTCCATA
	Tos17 LB	ATTGTTAGGTTGCAAGTTAGTTAAGA
Genotyping of T-DNA mutant rice	T-DNA OsPCS1 Fwd	GCAAGTCCGTGATACTGAGC
	T-DNA OsPCS1 Rev	GGATTAATACAACAAACCTCTCG
	POSTECH T-DNA RB	GCACCGTGGTAGTAAGAATGG
RT-PCR of *OsPCS1*	OsPCS1full/a Fwd	GACTTCCGCCACCATCTC
	OsPCS1 3'UTR Rev	CTTTTGCAAGGACACCAGCC
Real-time PCR of *OsPCSs*	OsPCS1full/a Fwd	GACTTCCGCCACCATCTC
	OsPCS1b/c Fwd	CATTGCATTGCATTTCAGAC
	OsPCS1full/b Rev	CATCTCTGCAACTCACTGTG
	OsPCS1a/c Rev	GCAACTCTGCACAAAAGCAC
	OsPCS2 Fwd	ATGGCGTCTAAACCAAGCAG
	OsPCS2 Rev	CGGAGACGAGGCTGAAGAAC
Real-time PCR and RT-PCR of *OsUBQ5*	OsUBQ5 Fwd	GAAGGAGGAGGAAATCGAAC
	OsUBQ5 Rev	CTTCACAGAGGTGATGCTAAGG

Lowercase sequences indicate additional sequences for restriction enzyme digestion.

Supplemental Table S2 Element concentrations and pH of the tested soil

Element concentration (mg kg^{-1} DW)	As	0.73 ± 0.03
	Cd	0.015 ± 0.001
	Fe	26.9 ± 2.2
	Mn	53.3 ± 2.4
	Cu	2.06 ± 0.10
	Zn	3.99 ± 0.08
pH (H_2O)		4.83 ± 0.03

Data represent mean with SD of 4 replicates.
